# Fast information and slow evidence in the large language models era

**DOI:** 10.1038/s41746-026-02909-7

**Published:** 2026-07-03

**Authors:** Yilan Wu, Ariel Yuhan Ong, Kuang Hu, Tien Yin Wong, Pearse A. Keane

**Affiliations:** 1 Institute of Ophthalmology, University College London, London, UK; 2 Beijing Visual Science and Translational Eye Research Institute (BERI), Beijing Tsinghua Changgung Hospital Eye Center, School of Clinical Medicine, Tsinghua Medicine, Tsinghua University, Beijing, China; 3 Moorfields Eye Hospital NHS Foundation Trust, London, UK; 4 Oxford Eye Hospital, Oxford University NHS Foundation Trust, Oxford, UK; 5 NIHR Biomedical Research Centre at Moorfields Eye Hospital NHS Foundation Trust, London, UK; 6 School of Biomedical Engineering, Tsinghua Medicine, Tsinghua University, Beijing, China; 7 Singapore Eye Research Institute, Singapore National Eye Centre, Singapore, Singapore

**Keywords:** Computational biology and bioinformatics, Health care, Medical research, Scientific community

## Abstract

LLMs accelerate medical information work, but speed of synthesis does not justify reliance. As T.S. Eliot asked, “Where is the knowledge we have lost in information?” Using a data-information-evidence-practice hierarchy, LLM outputs enter clinical setting as information and become evidence only through appraisal, validation, and contextual judgement. We highlight how LLMs can support evidence infrastructures, clarify evidence boundaries, and reshape clinical expertise to strengthen rather than replace evidence-based medicine.

## Introduction

Many clinicians and researchers have greeted large language models (LLMs) with a mix of enthusiasm and concern. On one hand, LLMs offer rapid, near-instant access to tailored summaries of complex information (“fast information”) and can assist in drafting everything from clinical notes to research papers and grant proposals. On the other hand, there are concerns that LLMs may flood the literature with rapidly produced, thinly supported concepts, manuscripts and proposals that mimic the language of evidence without underlying rigour^1^. However, both perspectives overlook a crucial context: LLMs are not an unprecedented challenge to medicine’s knowledge infrastructure but rather the latest iteration of a much older problem.

Long before the first LLM generated a clinical summary, medicine was already drowning in information. Evidence-based medicine (EBM) arose as a direct response to this problem in the 1990s, when clinicians were faced with over 2 million new biomedical articles annually, published across more than 20,000 journals^2^. Traditional approaches, such as narrative reviews, textbook updates, and individual expertise struggled to keep pace. EBM offered a systematic solution: structured methodologies for appraising evidence quality, hierarchies distinguishing strong from weak study designs, and specialised teams to distil primary research into guidelines that busy clinicians could apply at the point of care. The innovation was not simply to produce more information but to create explicit processes for transforming an unmanageable stream of fragmented information, diverse publications into safe, reliable, actionable evidence. EBM offered safe, practical and feasible clinical practice guidelines that withstood the test of time (“slow evidence”).

Three decades later, the flood of information has become a deluge. Over a million papers are added to PubMed annually^3^, and the gap between publication, synthesis, and clinical adoption continues to widen. LLMs enter this landscape offering a new layer of “fast information” processing capability^4^, providing help with literature gathering, summarisation, and personalisation tailored to the specific question, all delivered in seconds. LLMs function as accelerators for the information synthesis that EBM pioneered. However, by lowering barriers to both accessing and generating text, LLMs also intensify the very problem they promise to solve. The widespread concern about “AI fabrication”^5,6^ illustrates the core tension: we now have more information and words but not necessarily more knowledge and evidence.

In this Perspective, we ask: when LLM-supported outputs appear at different stages of the evidence workflow, what kind of evidential status are they? We introduce a “data - information - evidence - practice” hierarchy to contextualise where LLMs can operate safely, where they can expand unvalidated information, and what must remain grounded in clinical and methodological judgement. We argue that inclusion of LLM-produced information in the workflow is not itself sufficient to give it evidentiary status. Our aim is to clarify where LLM outputs can safely function as information, and where human methodological and clinical judgement remains essential for progression toward evidence and practice.

## Information overload, from EBM to LLM

For thirty years, EBM has influenced how clinicians make decisions in practice. Sackett and colleagues defined it as the “conscientious, explicit, and judicious use of current best evidence in making decisions about the care of individual patients”^7^. In practice, this means relying on systematically appraised evidence, often distilled into reviews and guidelines, rather than on individual authority alone. This division of labour allows the slow work of evidence synthesis to support the faster, practical decisions at the point of care. EBM, at its best, brings reliability to a world of constant change.

Yet the system shows signs of stress. Systematic reviews may already be out of date by the time they are published, and clinical guidelines can lag years behind new discoveries. Moreover, EBM has never been a complete description of how clinical decisions are made. There are many situations including rare diseases, complex multimorbidity, or ethically challenging questions in which rigour results simply do not exist^8–10^. In these moments, clinicians must draw on a blend of limited evidence, their own experience, and patient preferences. The link between evidence and clinical judgement is rarely as clear-cut as the textbooks suggest.

It is worth noting that the field of computational assistance in evidence synthesis pre-dated the foundation model era. For more than two decades before LLMs, natural language processing (NLP) tools automated aspects of information extraction, abstract screening^11^, and data capture from clinical text^12,13^. Those systems established proof-of-concept for machine-assisted evidence workflows and surfaced the same core challenges: accuracy degradation on classification tasks for controversial questions that remain relevant today.

This is the context in which LLMs contribute to lowering the barrier to information access and generation. Clinicians can now obtain draft summaries of guideline recommendations, topic overviews, or suggested reading lists that would previously have required multiple website searches and manual filtering^14^. In this sense, LLMs offer a new, faster layer of information synthesis that sits on top of existing evidence pipelines.

The growing use of LLMs is also influencing the landscape of evidence synthesis, with “AI-specific vocabulary footprint” increasingly appearing in published literature^15,16^. Systematic reviews and meta-analyses rely on the assumption that the primary literature mainly consists of genuine, methodologically sound studies. As mentioned by the Cochrane position statement of AI use in evidence synthesis, users are ultimately responsible for the downstream outputs^17^. If the literature used for these syntheses includes key claims without proper verification, even well-conducted reviews could be based on fragile foundations. This presents EBM with a dual challenge: managing the volume of information, while also verifying its authenticity and clinical validity. While LLMs are highly capable at synthesising information by connecting, reorganising, and summarising text at scale^18^, they cannot autonomously produce the validated evidence that medical decisions require. To navigate this landscape effectively, we need clearer conceptual distinctions between the types of information LLMs process, and the evidence that clinical practice demands.

## From data to evidence

To better understand the situation, it is useful to clearly distinguish between data, information, and the hierarchy of evidence^19,20^.

**Fig. 1:**
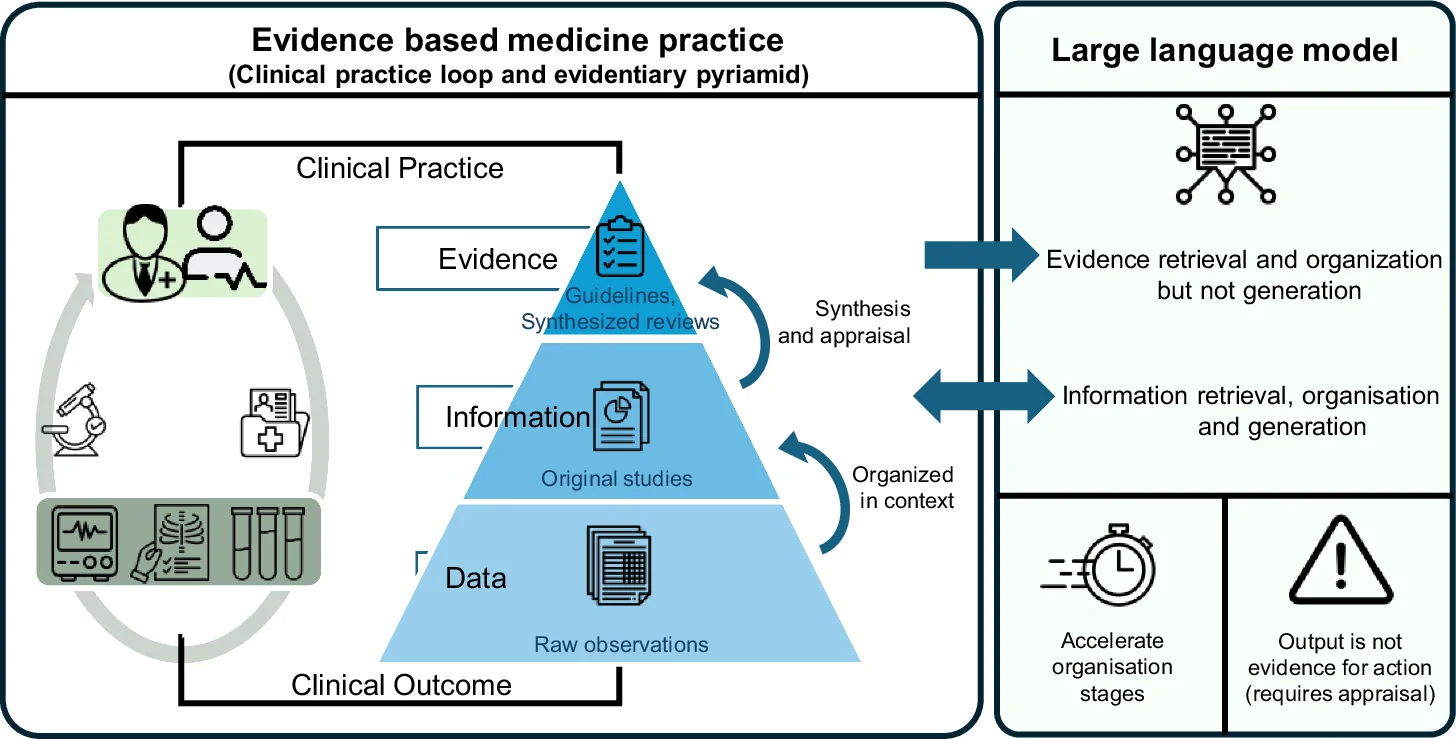
Layered process from data to practice. This conceptual figure matches LLMs within the evidentiary pyramid in clinical practice. In evidence-based practice, the clinical outcome can form data in the evidentiary pyramid, interpreted as information through original studies, and appraised to evidence, which then link back to the practice loop by informing clinical decision. LLMs can accelerate work at the levels of data and information by retrieving, organising or even generation, and in some cases can also assist with evidence retrieval and organisation. However, LLM outputs do not by themselves generate evidence for clinical action.

Data consists of raw observations, such as patient measurements, lab results, or study outputs. When organised and placed in context, for example, in a clinical report, data turns into information. This information transforms into evidence when it is aligned with methodological standards for conducting high quality research. Evidence can then be systematically synthesised into guidance for practice through meta-analyses, guidelines, and other EBM outputs. Evidence synthesis, in this sense, is a layered process: it uses methods of information synthesis such as searching, screening, extraction, and summarisation as inputs to human-led appraisal and quantitative synthesis (Fig. 1).

Consider diabetic retinopathy screening as an example, which begins at the data level with fundus photographs and demographic and clinical variables such as diabetes duration and HbA1c. When an algorithm or human grader interprets the images, labels microaneurysms and haemorrhages, and assigns a diagnosis, these data are transformed into information. Longitudinal cohorts and clinical trials then provide evidence based on this information about how often people with diabetes develop diabetic retinopathy, and show that early detection and treatment of sight-threatening retinopathy reduces the risk of vision loss. The current clinical recommendation that adults with diabetes should undergo regular fundus examinations is the final step, where evidence from many studies has been synthesised into actionable guidance for practice.

Recent work demonstrates the inclusion of LLMs across core EBM workflow tasks: pre-filtering titles and abstracts with performance approaching that of human reviewers^21,22^; full-text screening achieving human-comparable accuracy when relevant studies substantially outnumber irrelevant ones^22^; structured data extraction exceeding 98% accuracy on pre-specified elements from supplied text^23^; and PICO element recognition reaching around 80% across clinical domains^24^, further improved by knowledge-guided prompting^25^. End-to-end systems now cross the full arc from question decomposition through retrieval, screening, extraction, and draft recommendation generation, which substantially compressed the person-hours that systematic evidence synthesis has historically demanded^26^. These developments suggest meaningful acceleration in the earlier stages of evidence workflows, while leaving appraisal-dependent stages less reliably automated.

Yet each of these capabilities reveals a corresponding failure mode precisely located within the data–information–evidence hierarchy. PICO extraction at ~80% accuracy conceals substantially higher error rates for causal inference items and cross-disciplinary design elements^24^. Full-text screening that approaches human performance under optimal prompting degrades sharply when inclusion and exclusion criteria are balanced, approaching zero discriminative power in 1:1 datasets^22^, characteristic of genuinely contested clinical questions. And at the risk-of-bias and GRADE-appraisal steps, which are the transitions that validate movement from information to evidence, even the most capable systems reproduce superficial text that mimics appraisal of appraisal language. However, they do not perform the evaluative reasoning that gives that information its evidentiary quality^27,28^. Three more fundamental failure modes compound these problems. LLMs may generate information without underlying data, producing confident-sounding statements and citations that are fabricated, inaccurately quoted or unsupported^29,30^. They can amplify data without real information, contributing to a growing volume of homogenous or minimally novel papers without adding substantive insight^15,31^. Additionally, they may provide information without verification: even when summarising genuine sources, current systems rarely expose clear evidence chains, and struggle to signal uncertainty^29^. Thus, LLMs are rendering the evidential status of their outputs invisible to the reader.

Emerging architectural approaches address some of these failure modes. Retrieval-augmented generation (RAG) systems ground outputs in curated, verified sources rather than parametric memory, substantially reducing fabricated citations and producing actionable answers for 48% of queries with existing evidence, compared to under 5% for general-purpose LLMs under equivalent evaluation conditions^32^. Agentic systems may support exploratory analyses on real-world data in areas where published evidence is sparse, but such outputs still require methodological governance before they can inform evidence claims^32^. Nonetheless, these approaches converge on a deeper limitation: LLMs model statistical regularities in text rather than applying explicit evidence standards^33^. RAG-based systems retrieve evidence which is not equivalent to generating evidence as the evidential status of input does not automatically apply to the output. The evidence synthesis step, which requires adjudicating consistency across studies, assessing indirectness, and calibrating confidence, has yet to be perfomed^27,28^.

This is why recent workflow-participation findings sharpen rather than diminish the relevance of the data-information-evidence-practice hierarchy: they show that LLMs can produce outputs across multiple stages of evidence synthesis, but not that those outputs have acquired the evidential status of evidence for action. The distinction is reflected in frameworks such as GRADE (Grading of Recommendations Assessment, Development, and Evaluation), which characterises evidence quality along dimensions of risk of bias, inconsistency, indirectness, imprecision, and publication bias^34^. However, studies directly testing LLM performance on risk-of-bias assessment find domain-level accuracy of 57–70% against expert Cochrane judgements, leading to the conclusion that LLM assessments cannot yet replace human appraisal^17,35,36^. Thus, LLM-generated outputs across EBM workflows do not automatically confer a standard for clinical action.

## Reimagining evidence systems for the AI era

**Fig. 2:**
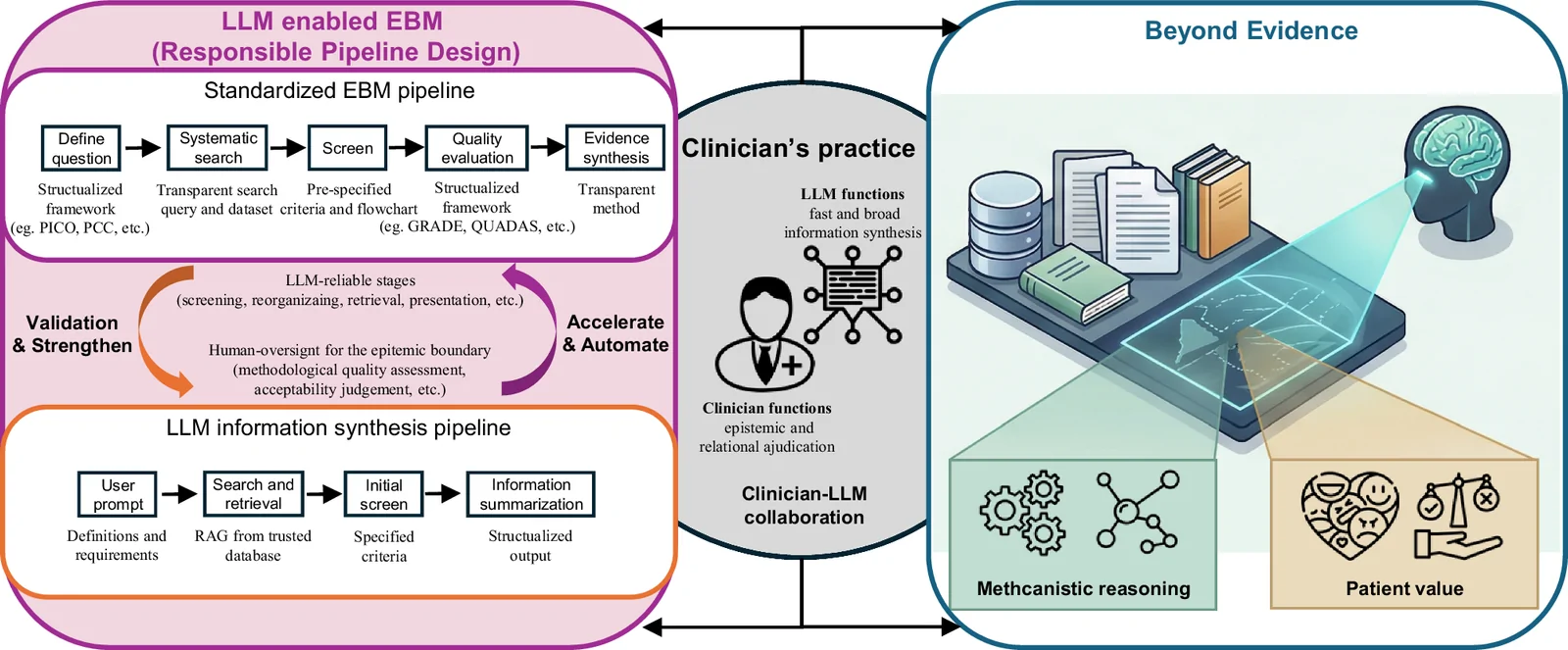
Potential methods for integrating LLMs into clinical practice. Large language models (LLMs) can potentially be integrated into clinical practice through mutual enhancement with evidence-based medicine (EBM), as shown in the left panel; by exploring beyond EBM, as shown in the right panel; and by through human-LLM collaboration with practicing clinicians, as shown in the middle panel.

Having established where LLMs operate within the “data-information-evidence” hierarchy, we can now address how they may be integrated into clinical practice. Three pathways emerge for productive LLM integration: treating them as tools within a mature evidence framework, using them to identify what current evidence fails to cover, and considering how human-LLM collaboration can improve performance (Fig. 2). We return to the DR vignette to illustrate each pathway concretely.

### From tools to evidence infrastructure

The preceding analysis identifies the data-to-information transition as where LLM contributions are most reliable, and the information-to-evidence transition as where appraisal judgement cannot yet be delegated^37,38^. Responsible pipeline design follows from that boundary directly. Three structural conditions must be satisfied. First, LLM contributions must be architecturally confined to the stages where they are evidentially reliable: those involving the reorganisation, retrieval, and structured presentation of existing information^39^. Second, the appraisal checkpoints that justify the transition from information to evidence include methodological quality assessment, applicability judgement, contextual synthesis. These must be operationally enforced as human functions, not merely recommended as best practice^17^. Third, the pipeline as a whole, rather than the model output in isolation, must be the auditable unit^40^: it is the end-to-end behaviour of the system, including its human governance steps, that either satisfies or fails to satisfy evidentiary status conditions.

In the DR vignette, these conditions translate into a concrete structural separation. PICO formulation, literature querying, abstract screening, data extraction, and result tabulation can be executed by the LLM component, operating quickly and at scale, with every output traceable to its source. More capable systems including agents extend the data-to-information transition to richer outputs without changing what the conditions require. At the point where the question becomes whether the retrieved evidence is methodologically sound, whether the trial populations generalise to this patient, and how evidence should be weighted against the patient’s values and local resource context, the pipeline yields to human appraisal. That boundary is not only a practical constraint of current systems, but reflects the governance functions required for evidentiary use in clinical medicine.

### Beyond evidence and EBM

Many clinical decisions occur at or beyond the boundaries where formal evidence exists. In these zones, clinicians must navigate limited studies, mechanistic reasoning, local experience, and patient values without standardised guidelines. The DR vignette can also illustrate this situation: annual screening for proliferative DR carries guideline-level evidence, yet evidence for optimal intervals in patients with early stable disease on novel agents, in resource-constrained settings, or across ethnically diverse retinal morphologies is substantially thinner. Decisions in these zones are made on information, not evidence.

Thus, it is valuable to use LLMs to traverse the published landscape to identify precisely where the information-to-evidence transition has not been made, and rendering that boundary explicit^41^. A useful example is OpenEvidence, which can retrieve and synthesise source-grounded publications and guideline material for clinicians, but the evidential status of those materials still depends on clinician appraisal of their quality, relevance, and applicability. The LLM does not itself generate evidence; rather, it organises information so that clinicians can judge what should count as evidence in the case at hand. In that sense, An LLM-assisted gap map with provenance and explicit uncertainty signalling can be more honest than a synthesised answer that projects false completeness across an uneven evidential terrain^42,43^.

This gap-mapping function connects to two further dimensions that formal trials rarely capture. The first is mechanistic reasoning: LLMs can integrate findings from basic science and clinical case reports to generate plausible hypotheses about the mechanism of action or therapeutic targets, as proposed in the description of “deep evidence-based medicine”, which integrates multimodal information and multiple dimensions of evidence beyond the traditional EBM hierarchy^44^. In the DR vignette, this might involve surfacing emerging evidence on GLP-1 receptor agonists and retinal vascular outcomes, which can be relevant to screening interval decisions, but not yet formalised into guideline-level evidence.

The second dimension is patient values. The trade-offs relevant to DR management, such as compliance burden versus detection sensitivity, or treatment anxiety versus reassurance benefit, are rarely documented even in clinical trials. LLMs have the potential to surface scattered information on patient preferences, convert it into a concise set of decision-related constraints to inform study design and evidence interpretation^45^.

Real-time linkage to living evidence infrastructure represents the next horizon for both functions. Systems connected to continuously updated literature feeds, trial registries, and regulatory databases can narrow the lag between evidence generation and clinical availability, which currently renders many systematic review conclusions outdated before publication^26^. LLMs can accelerate the data-heavy steps of horizon scanning, data coding, and gap-map drafting, allowing human effort to concentrate where methodological judgement is irreplaceable: scope definition, protocol development, and critical appraisal^41,42^.

### Clinical expertise, evidential literacy, and research culture in the LLM era

The integration of LLMs into clinical workflows raises questions about the evolution of clinical expertise. The concern that clinicians will be reduced to signing off on machine-generated decisions, misreads the change. As information becomes more accessible, the clinician’s role shifts toward interpreting and contextualisation, which aligns with the original intention of EBM, which was to educate clinicians in understanding and critically evaluating published literature^46^.

The centaur chess analogy is instructive^47^: human-model teams outperform either humans or engines alone because the human learns to direct the engine, about when to trust, when to override, when to request alternative lines. A mature LLM-enabled clinical workflow may look similar. LLMs perform fast, broad information synthesis, generating structured comparisons, and presenting competing options. Clinicians perform what remains irreducibly clinical: deciding what counts as relevant evidence for the patient, integrating risks with lived priorities, and managing uncertainty honestly. In this framing, accountability is not only about legal responsibility^17^. It also involves an evidential dimension, which means ensuring that decisions remain compliant with clinically validated information when available, and are transparently value-driven when evidence is thin.

The DR vignette clarifies which steps cannot be delegated without loss of evidentiary status: adjudicating indirectness (do the trial populations represent this patient’s demographic, comorbidity profile, and healthcare context?); assessing inconsistency across guidelines developed under different methodological assumptions; and translating evidence into contextualised action given the patient’s values, risk tolerance, and local resource reality. What changes is not the necessity of these functions but where human effort concentrates less on retrieval and initial synthesis but more on appraisal, adjudication, and the accountable final judgement that converts evidence into practice.

As agentic systems take on autonomous question formulation, search strategy design, and report generation, the human role shifts structurally from co-executor to governor^17^. This raises rather than reduces the evidential warrant requirement. A governor who cannot distinguish information from evidence at a pipeline checkpoint, and who cannot recognise whether an output has acquired evidentiary status or only resembles it, cannot exercise meaningful oversight of the system they are responsible for. Evidential literacy about the data-information-evidence-practice hierarchy is therefore a core professional competency for the AI era, and one that intensifies as automation increases distance from intermediate steps.

This vision carries a corresponding responsibility for research culture. As we move forward, it is worth remembering the value of “slow science”. Efficiency gains from AI must be reinvested into depth and rigour, not volume^48^, Incentive structures that reward publication count risk privileging AI-assisted repetitive output over the sustained methodological work that produces genuinely new evidence^49,50^. Evaluation frameworks need to value data integrity, reproducibility, methodological soundness, and demonstrable impact, qualities that simply text generation cannot easily fake, and that the data-information-evidence-practice hierarchy exists, ultimately, to protect.

#### Limitations

Several limitations of this analysis deserve acknowledgement. First, this is a conceptual Perspective rather than a systematic review; our claims are illustrative rather than exhaustively synthesised evidence. Second, the empirical base on LLM performance in evidence workflows is advancing rapidly, and specific performance figures cited here may be superseded by the time of publication. Third, this paper does not fully address the regulatory, legal, reimbursement, or clinical deployment questions that any real-world implementation would require; those are important practical dimensions, but they are distinct from the evidential questions we pursue here.

## Conclusion: evidence in the age of artificial intelligence

EBM forms the foundation of modern clinical practice, and the entry of LLMs into its infrastructure represents an inflection point that requires deliberate choices. Throughout this perspective, we have organised that challenge around the conceptual data-information-evidence-practice hierarchy. At the data and information levels, LLMs excel at retrieval, reorganisation, and rapid synthesis at scale. At the evidence level, the transition requires appraisal, methodological judgement, and validation that current systems cannot reliably perform. At the practice level, human accountability to patient context and values remains constitutive and irreplaceable. The hierarchy identifies the structural conditions that any responsible integration of LLM technology into clinical practice need to satisfy, including appraisal, methodological judgement, and contextual validation remain human functions at the evidence level, regardless of how capable retrieval and synthesis systems become.

Three conclusions follow from this analysis. First, workflow participation does not equal evidentiary status: an LLM output that crosses multiple stages of an evidence pipeline has not thereby acquired the evidential standing of evidence for action. Second, human governance becomes more important, not less, as systems become more agentic; the shift from co-executor to governor raises rather than relaxes the evidential literacy required of clinicians and institutions. Third, the value of LLMs extends beyond accelerating existing pipelines to an underused function: mapping the boundaries of evidence, and directing human effort toward the methodological work that evidence generation demands.

When LLMs are used within the hierarchy, and only where the conditions for evidentiary status are met, they can extend the reach of EBM, clarify its limits, and focus clinical expertise where it is hardest to replace. The LLM era will not weaken EBM; it will test whether the institutions and clinicians who depend on it can hold the line between information and evidence precisely when speed and volume most tempt them to blur it.
